# Validity of EQ-5D-5L in stroke

**DOI:** 10.1007/s11136-014-0834-1

**Published:** 2014-10-28

**Authors:** Dominik Golicki, Maciej Niewada, Julia Buczek, Anna Karlińska, Adam Kobayashi, M. F. Janssen, A. Simon Pickard

**Affiliations:** 1 Department of Experimental and Clinical Pharmacology, Medical University of Warsaw, Banacha 1b St, 02-097 Warsaw, Poland; 2 2nd Department of Neurology, Institute of Psychiatry and Neurology, Jana III Sobieskiego 9 St, 02-957 Warsaw, Poland; 3 Department of Medical Psychology and Psychotherapy, Erasmus MC, Erasmus University, PO Box 2040, 3000 CA Rotterdam, The Netherlands; 4 Department of Pharmacy Systems, Outcomes, and Policy, College of Pharmacy, University of Illinois at Chicago, 833 South Wood St, MC886, Chicago, IL USA

**Keywords:** EQ-5D-5L, Health-related quality of life, Patient-reported outcomes, Psychometrics, Stroke

## Abstract

**Purpose:**

To assess EQ-5D-5L (5L) validity in patients with acute stroke, in comparison with EQ-5D-3L (3L).

**Methods:**

Cross-sectional study of 408 patients during index hospitalization. We compared 5L and 3L in terms of feasibility, frequency of unique health states, ceiling effect and discriminatory power (informativity). We assessed construct validity in terms of known-groups validity and convergent validity of 5L dimensions with other stroke outcome measures.

**Results:**

The overall proportion of patients with acute stroke reporting ‘no problems’ with 3L—6.1 % was further reduced to 5.6 % with 5L (relative reduction of 8.2 %). The highest improvement in relative discriminatory power, when moving from 3L to 5L, was noticed in pain/discomfort and anxiety/depression dimensions (Shannon Evenness Index 0.91 for both 5L dimensions; relative increase 34.4 and 29.1 %, respectively). Known-groups validity tests confirmed prior hypotheses: Health state utilities were lower in following subpopulations—females, patients with high modified Rankin Scale (mRS) score, low Barthel Index (BI) or VAS score, patients with subarachnoid hemorrhage or intracerebral hemorrhage, and when proxy respondent was used. Convergence of EQ-5D-5L dimensions with mRS, BI and EQ VAS was improved or at least the same as for 3L dimensions.

**Conclusions:**

Results support the validity of the EQ-5D-5L descriptive system as a generic health outcome measure in patients with acute stroke, demonstrating some psychometric advantages in comparison with EQ-5D-3L.

## Introduction

Three level EQ-5D is, probably, the most widely used generic health status questionnaire in patients experiencing stroke [1, 2]. An extensive body of literature has been published, establishing its psychometric properties in stroke patients: reasonable construct, concurrent and discriminant validity, accuracy for predicting outcomes [3–5] and responsiveness in longitudinal studies [4, 6].

Recently, the EuroQol group has introduced new, five level, version of the EQ-5D (EQ-5D-5L) [7]. Janssen et al. [8], in multi-country study involving eight groups of patients with chronic conditions, demonstrated advantages of the new version: valid redistribution, reduced ceiling, improved discriminatory power and improved convergent validity (in comparison with the WHO-5 generic questionnaire). Several other validity studies in selected populations: Patients with chronic hepatic diseases [9], HIV/AIDS [10] and cancer [11, 12], has been conducted. Nevertheless, specific analysis concerning stroke patients is still lacking.

The aim of our study was to assess the validity of the EQ-5D-5L, in comparison with EQ-5D-3L, in acute stroke patients.

## Methods

### Patients

Adult patients with cerebral infarction, intracranial or subarachnoid hemorrhage (I63, I61 or I60, according to the ICD-10 classification) were included into a single center cross-sectional study. A diagnosis had to be confirmed by clinical and neuroimaging examinations. Patients had to be Polish language native speakers. Patients in coma were excluded. In case of aphasia or dementia, the survey was completed by a family member serving as a proxy respondent.

### Measures

The survey took place during index hospitalization (median 8 days since admission). The degree of disability due to stroke was assessed with the modified Rankin Scale (mRS) [2], physical performance with Barthel Index (BI) [13], and health-related quality of life with the EQ-5D-5L (5L) and EQ-5D-3L (3L) generic questionnaires and the visual analog scale (EQ VAS). Quality of life instruments were always presented in the fixed, mentioned above order, with no other questionnaires between the 5L and 3L. Paper and pencil versions were used. To obtain 3L index values, we used the Polish EQ-5D-3L value set based on the time trade-off valuation technique [14] and to obtain 5L index, we used Polish interim EQ-5D-5L value set estimated with official crosswalk methodology as developed by the EuroQol Group [15, 16]. The study protocol was approved by the local ethics committee and all participants gave informed consent before inclusion.

### Analysis

We compared 5L and 3L in terms of feasibility (proportion of missing answers), frequency of unique health states, ceiling effect (proportion of ‘no problem’ answers) and discriminatory power (informativity) [8, 9]. We assessed construct validity in terms of known-groups validity and convergent validity of 5L dimensions with 3L dimensions and other stroke outcome measures.

The proportion and level of logical inconsistencies in pairs of 5L and 3L answers was analyzed as described by Janssen et al. [17]. Inconsistent responses were scored from 1 to 3, according to the distance from the consistent level.

To assess discriminatory power, we calculated the Shannon Index (*H*′), which represents the absolute amount of captured informativity and the Shannon Evenness Index (*J*′), which reflects the rectangularity of a distribution regardless of the number of levels, as described elsewhere [8, 18]. When a measure reaches the evenness of the distribution (rectangularity), *H*′ approaches 1.58 (3L) or 2.32 (5L) and *J*′ approaches 1.0, indicating maximum informativity captured by the instrument.

Known-groups construct validity was tested for 5L and 3L indexes in regard to: age and sex, type of respondent (patient or proxy), stroke type according to ICD-10, stroke outcome according to mRS, BI and EQ VAS [19]. We hypothesized that utility will be lower: with increasing age, in females, when the patient would be unable to respond by himself and a proxy respondent would be used, in patients with subarachnoid hemorrhage or intracerebral hemorrhage [20]. We expected that utilities will follow stroke outcomes assessed by other instruments.

Convergent validity was assessed by examining the strength of association between 5L and 3L dimensions with mRS score, BI score and EQ VAS and by comparing 5L and 3L dimensions between themselves using Spearman’s rank correlation coefficient [19]. Strength of correlation was interpreted using the following criteria: absent (*r*_s_ < 0.20), poor (*r*_s_ = 0.20–0.34), moderate (*r*_s_ = 0.35–0.50) or strong (*r*_s_ > 0.50) [21]. We hypothesized that: (1) 5L dimensions will have stronger correlations with mRS, BI and EQ VAS than 3L dimensions, (2) 5L dimensions that relate to functioning—Mobility (MO), Self-Care (SC) and Usual Activities (UA)—will more strongly correlate with stroke outcome measures (mRS and BI) than 5L pain/discomfort (PD) and anxiety/depression (AD) dimensions and (3) related 5L and 3L dimensions will have stronger correlations with each other.

The study data were analyzed using StatsDirect ver. 2.8.0 statistical software.

## Results

From July 2009 to May 2010, 408 patients (51.5 % males), mean age 69.0 years were enrolled (Table 1).

**Table 1:** Demographic characteristics of studied population

Patients characteristics	
N	408^a^
Age, years,	
Mean (SD)	69.0 (12.9)
Range	23–98
Sex, F, *n* (%)	198 (48.5)
Stroke type (ICD-10), *n* (%)	
Subarachnoid hemorrhage (I60)	8 (2.0)
Intracerebral hemorrhage (I61)	39 (9.7)
Cerebral infarction (I63)	353 (87.4)
Stroke, not specified (I64)	4 (1.0)
mRS, *n* (%)	
0–2	206 (51.2)
3–4	130 (32.3)
5	66 (16.4)
Barthel Index, mean (SD)	70.6 (34.7)
Barthel Index, n (%)	
0–24	62 (15.4)
25–49	40 (9.9)
50–74	47 (11.7)
75–99	100 (24.8)
100	154 (38.2)
VAS, mean (SD)	49.5 (26.2)
VAS, *n* (%)	
0–24	78 (19.7)
25–49	82 (20.7)
50–74	159 (40.2)
75–100	77 (19.4)
EQ-5D-3L index, mean (SD)	0.528 (0.382)
EQ-5D-5L index, mean (SD)	0.526 (0.375)
Respondent, *n* (%)	
Patient	315 (77.2)
Proxy	93 (22.8)

^a^Missing data included: age (2 patients), ICD-10 (4), mRS (6), Barthel Index (5), VAS (12), EQ-5D-3L index (15), EQ-5D-5L index (12)

A total of 2.9 % 5L and 3.7 % 3L questionnaires had at least one missing answer, indicating good feasibility of both instruments in patients with stroke. For 5L, missing values ranged from 0.25 % in MO to 1.5 % in UA. The overall proportion of inconsistent 5L responses (in comparison with 3L responses) was 3.5 %, ranging from 2.2 % for MO to 5.0 % for UA and with 86 % of inconsistencies being level 1, as defined by Janssen et al. [17]. The proportion of patients reporting ‘no problems’ was 6.1 % for 3L and 5.6 % for 5L (in comparison with 38.2 % for BI, 5.0 % for mRS and 2.5 % for EQ VAS). The relative reduction of the ceiling effect in 5L comparing to 3L (8.2 %) was the highest in SC dimension (13.5 %), followed by MO (10.1 %), AD (9.1 %), UA and PD (6.2 %, both). Shannon Index and Shannon Evenness Index showed perfect informativity of 5L MO dimension (*H*′ = 2.31; *J*′ = 1.00) and nearly perfect informativity of 5L UA (*H*′ = 2.27; *J*′ = 0.98) and SC dimensions (*H*′ = 2.26; *J*′ = 0.97), in patients with stroke. Nevertheless, the highest improvement in informativity, when moving from 3L to 5L, was noticed in PD and AD dimensions (relative increase of 34.4 and 29.1 %; *J*′ = 0.91 for both 5L dimensions, respectively). The total number of unique health states was 213 for 5L (most frequent 11,111; *n* = 22) and 62 for 3L (most frequent 22,222; *n* = 92).

Results for known-groups construct validity are shown in Table 2. In general, the results confirmed our hypotheses: index-based scores were lower in females, patients with high mRS score, low BI or VAS score, patients with subarachnoid hemorrhage (I60) or intracerebral hermorrhage (I61), and when the patient was unable to respond by him/herself and a proxy respondent was necessary. The only unexpected result was a lower health utility in patients up to 60 years of age, comparing to 61–70 years group. Index-based scores were similar for both 5L and 3L.

**Table 2:** Known-groups construct validity: mean index-based scores of EQ-5D-5L and EQ-5D-3L (and 95 % confidence intervals) by patient characteristics

	EQ-5D-5L		EQ-5D-3L	
	*n*	Mean (95 % CI)	*n*	Mean (95 % CI)
Age (years)				
0–60	98	0.587 (0.523; 0.651)	95	0.595 (0.527; 0.663.)
61–70	103	0.623 (0.554; 0.693)	104	0.612 (0.542; 0.681)
71–80	112	0.461 (0.390; 0.531)	111	0.473 (0.405; 0.542)
>80	81	0.428 (0.335; 0.522)	81	0.422 (0.322; 0.523)
Sex				
Female	193	0.485 (0.428; 0.541)	189	0.485 (0.427; 0.543)
Male	203	0.565 (0.517; 0.614)	204	0.567 (0.518; 0.617)
Stroke type (ICD-10)				
I60	7	0.292 (−0.134; 0.719)	8	0.390 (0.016; 0.764)
I61	37	0.456 (0.309; 0.603)	35	0.399 (0.222; 0.576)
I63	345	0.539 (0.501; 0.578)	342	0.545 (0.506; 0.583)
Modified Rankin Scale				
5	65	−0.027 (−0.099; 0.044)	65	−0.027 (−0.098; 0.044)
4	55	0.291 (0.205; 0.377)	56	0.271 (0.181; 0.360)
3	70	0.576 (0.522; 0.630)	71	0.597 (0.550; 0.644)
2	112	0.698 (0.666; 0.730)	108	0.705 (0.668; 0.742)
1	68	0.824 (0.794; 0.855)	68	0.828 (0.793; 0.863)
0	20	0.865 (0.785; 0.946)	19	0.884 (0.829; 0.939)
Barthel Index				
0–50	107	0.069 (0.008; 0.130)	107	0.079 (0.017; 0.140)
55–95	134	0.610 (0.572; 0.649)	133	0.601 (0.557; 0.645)
100	150	0.783 (0.758; 0.808)	148	0.795 (0.771; 0.819)
EQ VAS				
0–24	76	−0.002 (−0.069; 0.066)	76	0.002 (−0.074; 0.779)
25–49	78	0.428 (0.362; 0.495)	79	0.437 (0.371; 0.502)
50–74	157	0.670 (0.637; 0.703)	156	0.674 (0.641; 0.708)
75–100	77	0.855 (0.829; 0.882)	75	0.856 (0.831; 0.880)
Respondent				
Patient	306	0.653 (0.623; 0.682)	303	0.650 (0.619; 0.682)
Proxy	90	0.096 (0.016; 0.176)	90	0.114 (0.031; 0.196)

Moderate to strong correlations were found between 5L and mRS, BI and EQ VAS, with a minimum of −0.37 between PD and BI, and a maximum of 0.79 between UA and mRS (Table 3). In all cases, convergence of 5L dimensions was improved or at least the same as 3L dimensions. EQ-5D-5L MO, SC and UA dimensions were more strongly correlated with mRS and BI, than 5L PD and AD dimensions. Convergence between related 5L and 3L dimensions (Table 3, cells in italics) was better than between unrelated dimensions.

**Table 3:** Convergent validity with stroke outcome measures (mRS and BI), EQ VAS and EQ-5D-3L dimensions; cells with related 5L and 3L dimensions marked in italics

Dimension	mRS		BI		EQ VAS		EQ-5D-5L dimensions	EQ-5D-3L dimensions				
	5L	3L	5L	3L	5L	3L		MO	SC	UA	PD	AD
MO	0.75	0.75	−0.74	−0.72	−0.74	−0.71	MO	*0.87*	0.78	0.74	0.51	0.44
SC	0.78	0.78	−0.77	−0.76	−0.75	−0.72	SC	0.79	*0.91*	0.80	0.38	0.42
UA	0.79	0.74	−0.74	−0.69	−0.76	−0.75	UA	0.77	0.82	*0.87*	0.37	0.42
PD	0.42	0.36	−0.37	−0.33	−0.55	−0.43	PD	0.47	0.49	0.50	*0.70*	0.48
AD	0.44	0.40	−0.43	−0.39	−0.54	−0.47	AD	0.46	0.48	0.51	0.49	*0.71*

*AD* anxiety/depression, *MO* mobility, *PD* pain/difficulty, *SC* self-care, *UA* usual activities, *mRS* modified Rankin Scale, *BI* Barthel Index

## Discussion

According to our best knowledge, this is the first study reporting specific data on the validity of the EQ-5D-5L in stroke patients. We confirmed construct validity of the instrument in terms of known-groups and convergence validity with other established stroke outcome measures.

Known-groups validity showed similar results for both 5L and 3L. Index-based scores were lower in hypothesized subpopulations. Studying known-groups validity, we were surprised by the lower health state utilities in patients up to 60 years of age, comparing to the next age group. These results can be explained by a higher proportion of individuals with subarachnoid or intracerebral hemorrhage—stroke types associated with worse outcome (29.3 % compared to 8.2 % in older age groups).

Furthermore, our results support convergence validity of EQ-5D-5L with other stroke measures. As expected, we found moderate to strong correlations between 5L dimensions and mRS, BI or VAS. All coefficients were slightly higher or at least the same for 5L, in comparison with 3L.

Our results are in general in line with these obtained by other authors in different populations [8, 9, 11]. EQ-5D-5L has shown some advantages in comparison with 3L: slightly better feasibility and some improvement in informativity (especially in PD and AD dimensions). In distinction to other studied populations, we have noticed rather small reduction of ceiling effect, both in terms of absolute and relative reduction. This can be partially explained by low level of ‘no problem’ in acute stroke population at baseline (about 6 %). From the other side, Janssen et al. [8] indicated some populations with low-baseline ceiling effect (rheumatoid arthritis patients) and substantial relative improvement (about 70 %).

Our study has some limitations. Quality of life questionnaires were administered in the fixed order. This could affect the proportion of missing answers, which was lower for 5L (administered first). The desirable solution would be to present instruments in a random order. Second, there were no other questionnaires presented between 5L and 3L. The risk is that memory effects may affect the comparison of these two versions.

Although, there is some evidence supporting the use of proxy respondents for 3L [22, 23], to our knowledge, this is one of the first studies that examines the validity of proxy respondents using the EQ-5D-5L. Further studies, also with longitudinal design, are needed to assess other psychometric characteristics in stroke patients, such as responsiveness to change and reliability of the instrument in terms of test–retest.

To conclude, evidence supports the EQ-5D-5L descriptive system as a valid generic health outcome measure in patients experiencing acute stroke, with some psychometric advantages in comparison with the EQ-5D-3L.
